# PROGRESSIVELY LOWERED STRESS THRESHOLD FOR BEHAVIORAL SYMPTOMS OF DEMENTIA: A DYNAMIC STRUCTURAL EQUATION MODEL

**DOI:** 10.1093/geroni/igac059.193

**Published:** 2022-12-20

**Authors:** Carolyn Pickering, Maria Yefimova, Danny Wang, Christopher Maxwell, Rita Jablonski

**Affiliations:** The University of Alabama at Birmingham, Birmingham, Alabama, United States; Stanford Health Care, Stanford, California, United States; The University of Alabama at Birmingham, Birmingham, Alabama, United States; Michigan State University, East Lansing, Michigan, United States; University of Alabama at Birmingham, Birmingham, Alabama, United States

## Abstract

The progressively lowered stress threshold model suggests that due to impairments in coping, persons living with dementia have a reduced threshold for stress and respond with more behavioral symptoms of dementia as stress accumulates throughout the day. While the propositions of the model have not been evaluated, this model serves as the basis of non-pharmacological interventions for behavioral symptom management aimed at modifying the environment to reduce stressors. These interventions have mixed success, which may be due to traditional longitudinal measurement models that don’t account for the dynamic temporal nature of behavioral symptoms. This paper evaluates the progressively lowered stress threshold conceptual model as an explanation for behavioral symptoms of dementia and tests several of its hypothesized propositions using an intensive longitudinal design. A sample of N=165 family caregivers completed brief daily diary surveys for 21 days (n=2841) reporting on behavioral symptoms of their care recipients. Dynamic structural equation modeling was used as the analytic technique to examine the impact of caregiver and care recipient environmental stressors on the diversity of behavioral symptoms of dementia (number of different symptoms) to account for the nested data structure and autoregressive relationships. Results show direct relationships between environmental stressors and diversity of behavioral symptoms of dementia that same day and the following day. Findings provide support for the progressively lowered stress threshold model. Further, findings suggest an extension to the conceptual model is warranted given evidence of an exposure/recovery trajectory and the lagged effects of stress exposure on behavioral symptoms of dementia presentation.

